# Relationship between risk factors for impaired bone health and HR-pQCT in young adults with type 1 diabetes

**DOI:** 10.3389/fendo.2023.1144137

**Published:** 2023-03-03

**Authors:** Etienne B. Sochett, Mary Dominicis, Reza Vali, Amer Shammas, Yesmino Elia, Rahim Moineddin, Farid Mahmud, Esther Assor, Michelle Furman, Steve K. Boyd, Nina Lenherr-Taube

**Affiliations:** ^1^ Department of Pediatrics, Division of Endocrinology, The Hospital for Sick Children, University of Toronto, Toronto, ON, Canada; ^2^ Department of Diagnostic Imaging, Division of Nuclear Medicine, The Hospital for Sick Children, University of Toronto, Toronto, ON, Canada; ^3^ Department of Family and Community Medicine, Faculty of Medicine, University of Toronto, Toronto, ON, Canada; ^4^ Department of Radiology, McCaig Institute for Bone and Joint Health, University of Calgary, Calgary, AB, Canada; ^5^ Division of Endocrinology, University Children’s Hospital Zürich, Zürich, Switzerland

**Keywords:** bone health, HR-pQCT, type 1 diabetes, young adults, vitamin D, HbA1c (A1C)

## Abstract

**Objective:**

In type 1 diabetes, risk factors associated with impaired bone health contribute to increased risk of fracture. The aim of this study was to (1): compare the high-resolution peripheral quantitative computed tomography (HR-pQCT) parameters of young adults with type 1 diabetes with those of healthy controls (2), identify sex differences, and (3) evaluate the association between diabetes and bone health risk factors, with HR-pQCT.

**Methods:**

This is a cross-sectional study in young Canadian adults with childhood onset type 1 diabetes. Z-scores were generated for HR-pQCT parameters using a large healthy control database. Diet, physical activity, BMI, hemoglobin A1C (A1C) and bone health measures were evaluated, and associations were analyzed using multivariate regression analysis.

**Results:**

Eighty-eight participants (age 21 ± 2.2 years; 40 males, 48 females, diabetes duration 13.9 ± 3.4 years) with type 1 diabetes were studied. Low trabecular thickness and elevated cortical geometry parameters were found suggesting impaired bone quality. There were no sex differences. Significant associations were found: Vitamin D (25(OH)D) with trabecular parameters with possible synergy with A1C, parathyroid hormone with cortical parameters, BMI with cortical bone and failure load, and diabetes duration with trabecular area.

**Conclusions:**

Our data suggests impairment of bone health as assessed by HR-pQCT in young adults with type 1 diabetes. Modifiable risk factors were associated with trabecular and cortical parameters. These findings imply that correction of vitamin D deficiency, prevention and treatment of secondary hyperparathyroidism, and optimization of metabolic control may reduce incident fractures.

## 1 Introduction

In adults with longstanding type 1 diabetes, poor bone quality and increased fracture risk are documented (1, 2). More recently, increased fracture risk was also reported in children with type 1 diabetes (3). Metabolic control, severe hypoglycemia, and delayed puberty have all been implicated (4, 5), as have hyperinsulinemia, autoimmune inflammation, altered muscle function and vitamin D deficiency (6). However, there remain significant gaps in the understanding of determinants and mechanisms underlying bone health impairment and increased fracture risk.

Skeletal health and future fracture risk in both children and adults are largely dependent on normal bone mass accrual in childhood and adolescence (7). This process is dependent on several factors that may be jeopardized in individuals with type 1 diabetes such as nutrition, physical activity, and a normal hormonal and glycemic milieu (8). Given that approximately 40% of peak bone mass accrues in the 4 years surrounding peak height velocity (9) and significant changes in bone microarchitecture occur during puberty (10), the immediate post pubertal window provides an opportunity to better understand the net impact on bone health in the population who developed type 1 diabetes during childhood.

Bone mineral density (BMD) using dual-energy X-ray absorptiometry (DXA) in adults, children, and adolescents with type 1 diabetes has shown mildly reduced values when compared with healthy controls (11–13). However, a more recent study found no difference in BMD across the lifespan of indivduals with type 1 diabetes when compared with healthy controls (14), implying that BMD may not explain the increased fracture risk seen in this population.

High resolution peripheral quantitative computed tomography (HR-pQCT) is a diagnostic tool that assesses bone quality in terms of microarchitecture and volumetric BMD (vBMD) of both cortical and trabecular bone compartments, as well as an estimate of bone strength, which can independently predict fracture risk (15, 16). Studies have identified parameters, such as total vBMD, trabecular vBMD, cortical thickness, trabecular thickness, trabecular number and trabecular separation as predictors of incident fragility fractures (16). Similar data are not yet available for young adults.

Even so, there are a limited number of published studies using HR-pQCT in type 1 diabetes. Studies in both adult (17) and children (18, 19) with type 1 diabetes have shown similar findings of reduced trabecular bone geometry in the radius and tibia, where studies in children also show an increase in trabecular separation (18) and an increase in trabecular inhomogeneity (18, 19). Decreased cortical bone geometry parameters have also been shown in both populations of patients with diabetes (17, 19, 20). Reduced estimated bone strength (failure load) was observed in children with diabetes when compared with healthy controls (19, 20). Lastly, skeletal microarchitecture was found to be altered in children with diabetes early in the course of disease and among those with higher average glycemia (20), with hemoglobin A1C (A1C) negatively correlated to bone microarchitecture parameters and estimated bone strength (19).

The aims of this study using HR-pQCT in young adults with type 1 diabetes were to (1): compare the bone microarchitecture, volumetric BMD, bone geometry and strength of young adults with childhood onset type 1 diabetes with those of healthy controls (2), identify any sex-specific differences, and (3) determine whether diabetes duration, A1C, BMI, bone health related laboratory measures and physical activity were associated with HR-pQCT measures.

## 2 Methods

### 2.1 Study population and study design

Eighty-eight young adults between the ages of 17 to 27 years with type 1 diabetes, who as adolescents had participated in the Adolescent Type I Diabetes Cardio-Renal Intervention Trial (AdDIT, EudraCT Number: 2007-001039-72, Trial Registration Number: ISRCTN91419926) (21) in Canada from 2009-2015, were recruited into a longitudinal, observational study evaluating cardio-renal-bone health in young adults with type 1 diabetes at the Hospital for Sick Children (SickKids) in Toronto, Canada. This report is a cross-sectional study of the bone health assessment completed from 2018-2019.

Inclusion criteria for this study were confirmed diagnosis of type 1 diabetes according to the Canadian Clinical Practice Guidelines, and participation in AdDIT (21). Exclusion criteria were the presence of any significant medical or surgical disorders, such as eating disorders, untreated thyroid disease, celiac or Crohn’s disease; a history of delayed puberty; oligomenorrhea (fewer than 6 periods per year) or secondary amenorrhea; familial history of multiple fractures (> 3 long bone fractures); and other factors that might negatively impact bone health, including systemic glucocorticoid use, immobility, or BMI <18.5 kg/m^2^. Data was collected for diet, physical activity, and life-time fracture history for the purposes of identifying those participants who might have significant deviations from the reference range and who therefore would meet the exclusion criteria.

This study was approved by SickKids Research Ethics Board. All participants provided informed consent to participate in this study. The study was conducted in accordance with the Declaration of Helsinki.

### 2.2 Demographic data and clinical characteristics

Demographic characteristics including age, sex, and ethnicity was collected from each participant. History of concomitant medications, smoking status, significant family and fracture history (collected by questionnaire), as well as diabetes details including disease duration, daily total insulin dose (units/kg/day), and route of administration were also collected. Height was measured using a wall-mounted stadiometer and weight was measured by an electronic scale. BMI was calculated using these measurements.

Pubertal status was previously assessed in AdDIT using a Tanner staging self-reported questionnaire (22) to confirm sexual maturity and the absence of delayed puberty. In 5 participants, it was unclear whether sexual maturity had been reached at the last AdDIT visit. In these 5 participants, normal hypothalamic-pituitary-gonadal axis was confirmed at study entry with measurement of LH, FSH, estradiol and testosterone respectively. Female participants were questioned regarding frequency of menstruation.

### 2.3 Physical activity

Moderate to vigorous physical activity (MVPA) and electronic screen time for each participant was calculated using the Physical Activity Characteristics Questionnaire derived from the Canadian Health Measures Survey (CHMS) (23), and analyzed using Canadian Physical Activity Guidelines (24).

### 2.4 Dietary assessment

Dietary intake for a single typical day was obtained at study entry. A trained research assistant (RA) collected this information using a structured and scripted telephone interview. Information pertaining to portion size estimates, recipes, brands, and food preparation methods were documented. To improve portion size estimates, a hand portion guide was provided to participants for reference and household measures and weights were commonly referred to when discussing the foods. Dietary intake collection from each participant was reviewed by a registered dietician (RD) which was then entered into the web-based Automated Self-Administered 24-hour Dietary Assessment Tool (ASA24), Canadian Version (2018). Foods not available on ASA24 were entered manually. Dietary calcium, vitamin D, and magnesium were calculated and reported as daily intake and % Dietary Reference Intakes (DRI) (25, 26).

### 2.5 Biochemical investigations

Fasting blood and urine specimens were collected at baseline. The baseline visit occurred over a 12-15 month period. with no predominance of sample collection related to season.

% A1C, serum lipids, creatinine and urinary albumin, calcium and creatinine were measured in the Department of Pediatric Laboratory Medicine at SickKids using standard laboratory methods.

Early morning urine samples were collected during three consecutive days. Urinary albumin/creatinine ratio (ACR) was calculated from either the mean value of the three independent collections or from a blended sample.

Serum calcium (Ca^2+^), phosphate (PO_4_), and magnesium (Mg) were measured in the Department of Laboratory Medicine & Pathobiology, Toronto General Hospital, University Health Network in Toronto, Canada using standard laboratory measures. In the same laboratory, bone-specific alkaline phosphatase (bALP) was measured using ELISA plate reader on Molecular Devices SpectraMax Plus (using Tandem-R Ostase Immunoradiometric Assay) and serum cross-linked C-telopeptide (CTX) was measured using an immunoassay test on a Diagnostics e411 analyzer (Roche, Elecsys beta CrossLaps serum assay). 25-hydroxy vitamin D (25(OH)D), and intact parathyroid hormone (PTH) were measured using the Abbott Alinity i immunoassay system. CV for 25(OH)D: (20.20 nmol/L- 6.71%, 33.81 nmol/L- 5.57%, 83.33 nmol/L- 4.60%: CV for PTH: 4.4 pmol/L -5.46%, 33.5 pmol/L- 4.97, 108.5 pmol/L- 5.05%). Determination of Vitamin D sufficiency, insufficiency and deficiency was based on the Global Consensus Recommendations on Prevention and Management of Nutritional Rickets (25).

### 2.6 High-resolution peripheral quantitative computer tomography imaging

The non-dominant radius and tibia were scanned on a HR-pQCT scanner (XtremeCT II; Scanco Medical AG, Brüttisellen, Switzerland). The forearm and leg were immobilized in a carbon fiber cast. An anteroposterior scout projection of the scan site was acquired for positioning of the tomographic acquisition. A reference line was placed on the plateau of the distal radius or distal tibia. The scan started 9 mm and 22 mm for the radius and tibia, respectively, from the reference in the proximal direction, and spanned 10.2 mm in length. Images were reconstructed using an isotropic resolution of 60.7 μm (27), thus resulting in a stack of 168 parallel HR-pQCT (27, 28) slices. Total scan time was 2.0 min, with each acquisition resulting in effective dose of approximately 3 µSv. All scans were graded once by the same two technologists with regard to subject motion using the 5-level motion grading scale (best score as 1, worst score as 5) (27). At the time of scanning, if motion artifacts with a score of three or more were observed, then as per recommendations, the scan was repeated. After repeat, no scans were graded level 4 (severe motion artifacts) or 5 (extreme motion artifacts), and therefore no scans were excluded.

### 2.7 HR-pQCT image analysis

All scans were evaluated using the standard patient image evaluation protocol that was provided by the manufacturer, and previously described (29). First, the periosteal contour was automatically derived and manually altered by the same two technologists when contours visually deviated from the periosteal boundary. The endocortical contour was automatically created using a series of automatic morphological operations to separate the trabecular and cortical volumes of interest resulting in the uncorrected contour (AUTO method). Then, when the contour visually deviated from the apparent endocortical margin, it was manually corrected (S-AUTO method).

Standard morphologic analysis was used to measure volumetric BMD for total (TtBMD; mg HA/cm^3^) and trabecular (TbBMD; mg HA/cm^3^) bone, as well as trabecular number (TbN; mm^-1^), separation (TbSp; mm), and thickness (TbTh; mm) (30). An automated segmentation algorithm was used to obtain total and cortical cross-sectional areas (TtAr, CtAr, mm^2^), cortical volumetric BMD (CtBMD; mg HA/cm^3^), cortical thickness (CtTh; mm) and cortical porosity (CtPo; %) (29, 31).

FE analysis was applied to HR-pQCT images to estimate bone strength. As previously reported, FE meshes from the 3D HR-pQCT images were created using the voxel conversion approach. Then, uniaxial compression on each radius section was stimulated up to 0.7% strain. A single homogenous tissue modulus of 8,748 MPa and a Poisson’s ratio of 0.3 were applied to all elements. A custom FE solver (FAIM, version 8.0, Numerics88 Solutions, Calgary, Canada) was used for this analysis (27).

HR-pQCT parameters expressed as Z-scores were generated from a normative HR-pQCT database covering both sexes across the adult lifespan at the distal radius and distal tibia (www.normative.ca) (28, 32). The ethnicity distribution and BMI (our cohort: 24.7 kg/m^2^ (male) and 27.5 kg/m^2^ (female); normative cohort: 26.8 kg/m^2^ (male) and 26.1 kg/m^2^ (female)) was similar to the study cohort and their ages were well within the normative age range (32).

### 2.8 Statistical analysis

Descriptive statistics were calculated for demographic, clinical, and laboratory test variables. Mean, standard deviation (SD), and range (minimum and maximum values) were provided for continuous variables. Frequencies and percentages were calculated for categorical variables. Participant HR-pQCT Z-scores were plotted on the HR-pQCT normative database to determine the distribution of the results relative to the normative dataset. In the absence of studies for HR-pQCT Z-scores that indicate a measure to be increased or decreased, we followed the recommendations provided by Peacock et al. (33). This review suggested that when a primary outcome is continuous and the clinically meaningful difference is uncertain, statisticians and researchers may express the difference to be detected as a multiple of the SD. Using this standardised effect size approach, calculated as the difference in the means divided by the SD at baseline, a cut-off of ± 0.8 is described as a large effect size. Z-scores above +0.8 were therefore defined as high and Z-scores below -0.8 were defined as low in comparison with subjects in the normative database. Females and males were compared using the Wilcoxon unpaired two-sample t-test for continuous variables given that the data was not normally distributed. Pearson’s chi-square test was used for categorical variables.

The association between individual HR-pQCT Z-scores and BMI, type 1 diabetes duration, mean MVPA, mean 10-year A1C, 25(OH)D, PTH, serum calcium, CTX, and bALP were assessed using multivariable linear regression. These variables were selected from the literature as either known or likely to be associated with bone health. Variables not included in the multivariable regression association were tested using univariable regression. To investigate the association between BMI and cortical HR-pQCT parameters, analysis of variance (ANOVA) was used, with the cohort being divided in three groups: 18.5-24.9 kg/m^2^, 25.0-29.9 kg/m^2^, and > 30.0 kg/m^2.^

Results are reported as beta values, with p values < 0.01 considered significant, to account for multiple comparison. Results with p values > 0.01 and < 0.5 are provided in the text and in regression analysis tables to accomodate for the possibility of a type 1 error, but are not used in the interpretation of the results. All analyses were performed using SAS 9.4 software (SAS Institute Inc., Cary, N.C.)

## 3 Results

### 3.1 Clinical characteristics

The demographic and clinical characteristics of 88 young adults with type 1 diabetes (mean age: 21 ± 2.2 years; 40 males, 48 females) are shown in **Table 1**. Mean diabetes duration was 13.9 ± 3.4 years. Mean BMI was 26.2 ± 5.5 kg/m^2,^with 28% of the cohort being either overweight (33% female, 22% males) or obese 18% (22% female, 12% male). Mean BMI in females was significantly higher than in males (27.5 and 24.7, p=0.03). The cohort was predominantly Caucasian and non-smokers. Mean ± SD % A1C from 8 visits over the preceding 10 years was 8.18 ± 1.03.

**Table 1:** Demographics and clinical characteristics of participants with type 1 diabetes (T1D).

	Study Population	Reference Range
Number (N)	88	
Sex		
Male	40 (45.45%)	
Female	48 (54.55%)	
Age (years)	21.29 ± 2.16	
Height (cm)		
Male	177.04 ± 8.11	
Female	164.37 ± 6.27	
Weight (kg)		
Male	77.42 ± 13.60	
Female	74.51 ± 17.58	
T1D Duration (years)	13.91 ± 3.42	
Daily Total Dose of Insulin (units/kg/day)	0.82 ± 0.25	
Insulin Regimen		
Injection	36 (40.91%)	
Pump	52 (59.09%)	
HbA1c (%, mmol/mol) Study entry % mmol/mol > 10 years % mmol/mol	8.20 ± 1.46 66.11 ± 16.01 8.18 ± 1.03 65.89 ± 11.24	lt; 6% < 42mmol/mol
BMI (kg/m^2^)		18.5-24.9 25.0-29.9 ≥ 30
Male	24.70 ± 4.15	
Normal	22.25 ± 1.57	
Overweight	27.08 ± 1.36	
Obese	33.17 ± 2.65	
Female	27.52 ± 6.10	
Normal	22.36 ± 1.04	
Overweight	27.91 ± 1.12	
Obese	36.79 ± 4.53	
Ethnicity		
White	62 (70.45%)	
Black	7 (7.95%)	
Asian	10 (11.36%)	
Other	9 (10.23%)	
Smoking Status		
No	85 (96.59%)	
Yes	3 (3.41%)	

Data are expressed as n (%) and/or mean ± SD.

The cohort had experienced normal pubertal development, except for 5 subjects who had a mild delay in puberty. All were sexually mature at the time of study. Approximately 40% had a history of one or more lifetime fractures, with most fractures being peripheral. Twenty-one participants experienced 1 fracture, 9 participants experienced 2 fractures, and 1 participant had 3 fractures.

No significant differences were identified between the males and females in age, ethnicity, diabetes duration, or % A1C. Total daily insulin dose was similar in males and females. Serum creatinine was within the reference range, although significantly higher in males than females (66.8 and 53.9 umol/l respectively, p=0.001) (**Table 2**). Mean ± SD urinary ACR and eGFR were 1.38 ± 2.77 mg/mmol and 128.83 ± 11.0 ml/min/1.73 m^2^, respectively, for both male and female combined.

**Table 2:** Biochemical measures of calcium, vitamin D and bone metabolism.

Variable	n (%) or mean ± SD	Reference Range
Biochemical Measures		
Total Number (N)	88	
Serum Ca^2+^ (mmol/L)	2.38 ± 0.09	2.20-2.62
Serum PO^4^ (mmol/L)	1.39 ± 0.20	0.80-1.40
Serum Mg (mmol/L)	0.76 ± 0.06	0.70-1.10
25(OH)D (nmol/L)	61.13 ± 34.08	
Sufficient	49 (55.68%)	>50
Insufficient	26 (29.55%)	30-50
Deficient	13 (14.77%)	<30
PTH (pmol/L)	5.52 ± 1.92	2.0 – 9.4
bALP (ug/L)	22.19 ± 14.54	
Male	27.83 ± 18.87	8.2-32.8^‡^
Female	17.48 ± 6.80	5.9-30.5^‡^
CTX (ng/mL)	0.57 ± 0.31	
Male	0.70 ± 0.35	0.238-1.019
Female	0.45 ± 0.21	0.148-0.967
Creatinine (µmol/L)		
Male	66.9 ± 10.61	51-89
Female	54.36 ± 8.17	40-69
Urine Calcium : Creatinine Ratio	0.20 ± 0.19	<0.14
Dietary Intake		
Total Number (N)	79	
**Daily** Calcium Intake		163-1600^*^
Calcium (mg)^†^	807.3 ± 680.62	
% Calcium DRI	80.7 ± 68.1%	
**Daily** Vitamin D Intake		4-645^*^
Vitamin D (IU)	341.81 ± 548.32	
% Vitamin D DRI	57.4 ± 91.9%	
**Daily** Magnesium intake		70-984^*^
Magnesium (mg)	312.38 ± 151.46	
% Magnesium DRI	87.4 ± 42.5%	

Data are expressed as n (%) or mean ± SD.

^†^including supplements.

^‡^95% reference interval (RI), ages: 25-29.9.

^*^ intake range.

Participants reported a mean of 55.8 ± 39.6 min/day of MVPA, which is higher than that of 31 minutes per day for 18-39-year-old healthy individuals reported in the 2017 Canadian Health Measures Survey (34). A mean of 71.3 ± 36.1 minutes/day of screen time including use of computers, tablets, phones, and TV was recorded.

Daily dietary intake measures and related biochemical measures are presented in **Table 2**. The mean daily intake, range and DRI, respectively, for relevant nutrients were: calcium 807 mg (range 163-1600 mg), DRI 80.7%; vitamin D 341.8U (4-645 IU daily), DRI 57.4%; magnesium 312.4 mg (70–984), DRI 87.4%; phosphate 1288.6 mg daily (370-1815 mg) DRI 184.1%. Mean daily protein intake was 1.1 g/kg (0.31-3.7 kg; 21-270 g/day). Mean serum Ca^2+^ was within the reference range, while mean serum PO_4_ (1.39 ± 0.20 mmol/L) was at the upper end of the reference range. 44% of the cohort was either vitamin D insufficient (n=26) or deficient (n=13). PTH levels were mainly within the reference range of 2.0-9.4 pmol/L. It is recognized that different immunodiagnostic ostease assays may provide different reference ranges for bALP. However, using the 95% RI for bALP provided from the age- and sex-specific reference ranges of the immunodiagnostic assay (35), our subjects were well within this reference range. CTX levels were also within the reference range.

### 3.2 HR-pQCT measures compared with a normative database

**Figure 1:**
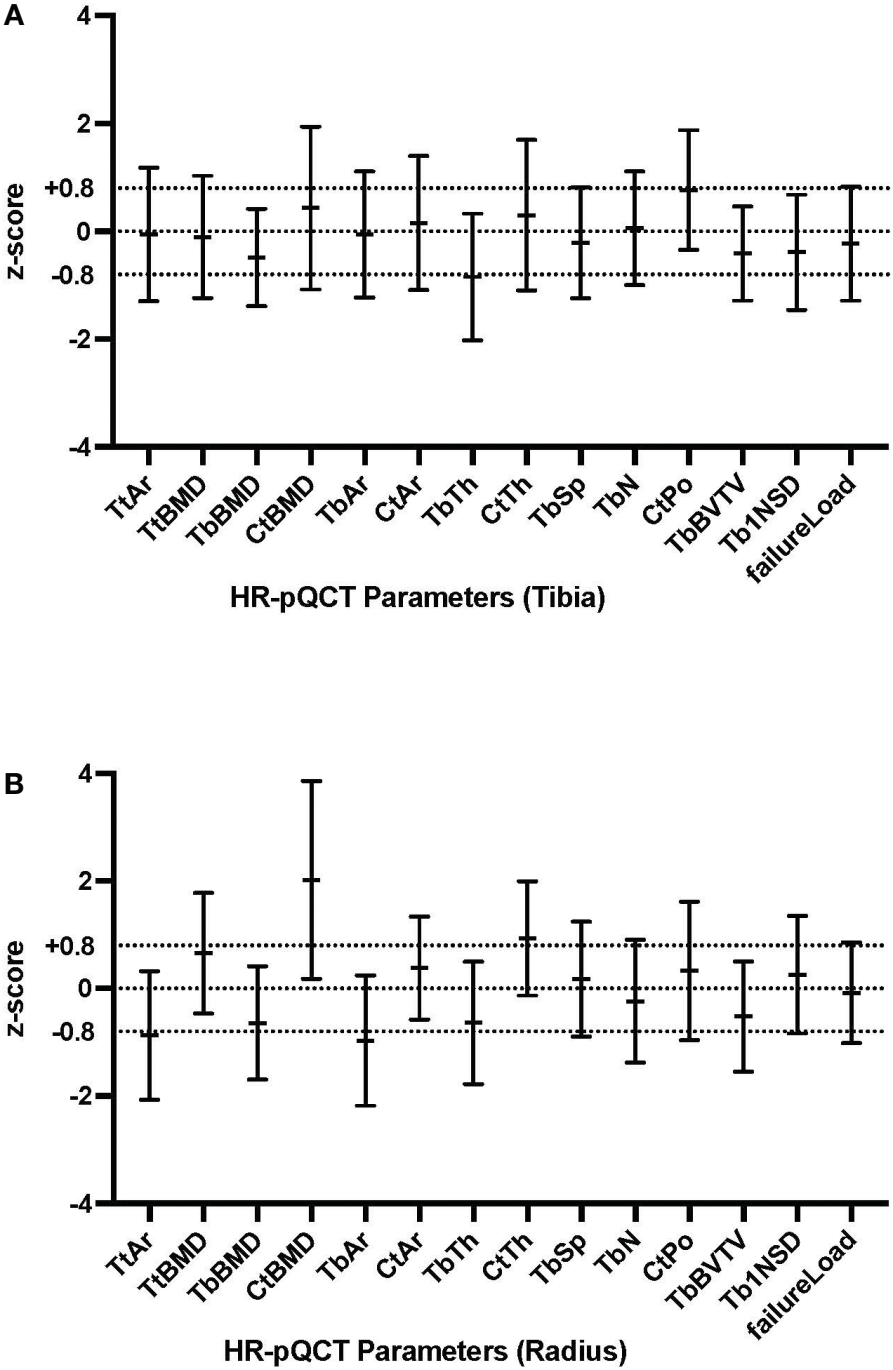
HR-pQCT parameters compared to Normative for tibia **(A)** and radius **(B)**.

HR-pQCT Z-scores for tibia and radius are presented in **Figure 1**. At the radius, cortical BMD (Z-score + 2.02) and cortical thickness (Z-score +0.93) were high and total area (Z-score -0.87) and trabecular area (Z-score -0.97) were low, in comparison with healthy controls. Trabecular thickness (Z-score -0.64) and trabecular BMD (Z score -0.64) were at the lower end of the defined range. At the tibia, trabecular thickness (Z-score -0.85) was low. The estimated bone strength (failure load) was similar to healthy controls and no sex-specific differences were found in any HR-pQCT Z-scores. Raw data of HR-pQCT measures are summarized in **Supplementary Table 1**.

### 3.3 ANOVA:cortical HR-pQCT measures and BMI

As demonstrated in **Supplementary Table 3**, BMI was found to be significantly associated with a number of cortical parameters: cortical area in both the radius (p=0.0159) and tibia (p=0.0001), both of which were in the defined Z-score normal range; cortical thickness in the tibia(p=0.0148), which is in defined normal Z-score range; and cortical porosity in the tibia (p=0.0034), with a borderline high Z-score, as shown in **Figure 1**. BMI was not associated with cortical vBMD (high Z-score).

### 3.4 Multivariate regression

The significant associations (p<0.01) between HR-pQCT measures, expressed as Z-scores, and diabetes and bone health-related measures are summarized in **Table 3** (radius) and **Table 4** (tibia). BMI was significantly associated with cortical area and failure load for both tibia (p<0.001 and p<0.001), and radius (p=0.002 and p <0.001), respectively.

**Table 3:** Regression analysis of HR-pQCT Z-scores of radius and relevant variables.

HR-pQCT Variables	Radius							
	BMI		T1D DUR		PTH		25(OH)D	
	β	*P*	β	*P*	β	*P*	β	*P*
TtAr	0.0650	**0.013^‡^**	-0.0774	**0.034^‡^**	-0.0327	0.630	0.0007	0.857
TtBMD	0.0023	0.926	0.0600	0.095	-0.1098	0.104	0.0021	0.569
CtBMD	-0.0180	0.638	0.0754	0.165	-0.2980	**0.004^†^**	-0.0042	0.450
CtAr	0.0631	**0.002 ^†^**	0.0233	0.393	-0.1620	**0.002^†^**	0.0016	0.568
CtTh	0.0246	0.308	0.0684	**0.046^‡^**	-0.1478	0.023	0.0011	0.747
CtPo	0.0282	0.377	0.0124	0.781	0.0249	0.765	0.0015	0.732
TbBMD	0.0061	0.803	-0.0239	0.490	-0.0226	0.728	0.0063	0.077
TbAr	0.0473	0.081	-0.0913	**0.018^‡^**	0.0103	0.886	0.0004	0.918
TbTh	0.0123	0.655	0.0348	0.369	-0.0319	0.661	0.0025	0.525
TbSp	0.0137	0.576	0.0520	0.134	0.0051	0.937	-0.0083	**0.020^‡^**
TbN	-0.0075	0.774	-0.0463	0.211	-0.0088	0.899	0.0096	**0.012^‡^**
TbBVTV	0.0083	0.727	-0.0228	0.497	-0.0120	0.849	0.0055	0.109
Tb1NSD	0.0197	0.434	0.0535	0.133	-0.0131	0.844	-0.0081	**0.027^‡^**
Failure Load	0.0681	**<0.001^†^**	-0.0001	0.997	-0.1279	0.011	0.0007	0.788

Multivariate linear regression was performed to assess associations between HR-pQCT Z-scores, and diabetes and bone health risk factors including BMI, T1D duration, MVPA, HbA1c, 25(OH)D, PTH, serum calcium, CTX, and bALP. Only those risk factors that were found to be statistically significant or borderline significant with HR-pQCT parameters are shown in the table above. Estimated parameters that show the amount of change in HR-pQCT Z-scores for one unit increase of covariates and p-values are reported.

^†^A p-value < 0.01 was considered statistically significant.

^‡^A p-value of < 0.05 and > 0.01 is borderline significant.

All analyses were performed using SAS 9.4 software (SAS Institute Inc., Cary, N.C., USA).

**Table 4:** Regression analysis of HR-pQCT Z-scores of Tibia and relevant variables.

HR-pQCT Variables	Tibia											
	BMI		T1D DUR		PTH		25(OH)D		HbA1c		BALP	
	β	*P*	β	*P*	β	*P*	β	*P*	β	*P*	β	*P*
TtAr	0.0485	0.075	-0.0969	**0.012^‡^**	0.0324	0.651	-0.0012	0.751	0.0766	0.567	-0.0039	0.803
TtBMD	0.0323	0.187	0.0552	0.110	-0.1348	**0.039^‡^**	0.0025	0.468	0.1172	0.332	-0.0151	0.291
CtBMD	0.0079	0.786	0.0737	0.074	-0.1091	0.159	-0.0070	0.094	-0.2534	0.081	-0.0205	0.229
CtAr	0.1129	**<0.001^†^**	0.0180	0.594	-0.1559	**0.016^‡^**	-0.0018	0.603	0.0185	0.876	-0.0317	**0.026^‡^**
CtTh	0.0703	**0.023^‡^**	0.0763	0.078	-0.1550	0.058	-0.0007	0.869	0.1179	0.435	-0.0177	0.321
CtPo	0.0264	0.316	0.0122	0.742	0.0047	0.946	0.0033	0.381	0.1613	0.217	-0.0127	0.408
TbBMD	0.0273	0.171	-0.0250	0.374	-0.0909	0.087	0.0050	0.084	0.2044	**0.041^‡^**	-0.0081	0.485
TbAr	0.0348	0.176	-0.1031	**0.005^†^**	0.0696	0.306	-0.0004	0.907	0.0512	0.686	-0.0016	0.913
TbTh	0.0588	**0.021^‡^**	0.0482	0.173	-0.1290	0.054	-0.0069	0.055	-0.0871	0.482	-0.0314	**0.034^‡^**
TbSp	0.0056	0.800	0.0427	0.174	0.0241	0.683	-0.0100	**0.002^†^**	-0.2797	**0.013^‡^**	-0.0100	0.440
TbN	0.0067	0.768	-0.0400	0.215	-0.0207	0.732	0.0099	**0.003^†^**	0.2623	**0.022^‡^**	0.0090	0.499
TbBVTV	0.0327	0.093	-0.0267	0.328	-0.0780	0.130	0.0042	0.132	0.1697	0.079	-0.0095	0.400
Tb1NSD	0.0233	0.318	0.0300	0.360	0.0124	0.841	-0.0105	**0.002^†^**	-0.2926	**0.013^‡^**	-0.0160	0.242
Failure Load	0.0935	**<0.001^†^**	-0.0424	0.139	-0.0884	0.101	-0.0007	0.801	0.0874	0.383	-0.0299	**0.013^‡^**

Multivariate linear regression was performed to assess associations between HR-pQCT Z-scores, and diabetes and bone health risk factors including BMI, T1D duration, MVPA, HbA1c, 25(OH)D, PTH, serum calcium, CTX, and bALP. Only those risk factors that were found to be statistically significant or borderline significant with HR p QCT parameters are shown in the table above. All other associations are summarized in the supplementary table S2. Estimated parameters that show the amount of change in HR-pQCT Z-scores for one unit increase of covariates and p-values are reported.

^†^A p-value < 0.01 was considered statistically significant.

^‡^A p-value of < 0.05 and > 0.01 is borderline significant.

All analyses were performed using SAS 9.4 software (SAS Institute Inc., Cary, N.C., USA).

At the radius, PTH was negatively and significantly associated with cortical area and cortical vBMD (p=0.002 and p=0.004, respectively) and showed borderline significance with cortical thickness (p=0.023) and failure load (p=0.011), both directionally negative. At the tibia, there was borderline significance between PTH and total vBMD and cortical area (0.039 and 0.016, respectively, both directionally negative). To further explore a potential interaction between PTH and 25(OH)D that could help explain these findings, 3-dimensional plots were constructed for these parameters. 25(OH)D levels showed either minimal or no nonsignificant interaction with PTH.

At the tibia, 25(OH)D was negatively and significantly associated with trabecular separation (p=0.002) and trabecular inhomogeniety standard deviation (p=0.002), and positively and significantly associated with trabecular number (p=0.003). There was a borderline significant association between mean 10-year A1C (8.18 +/- 1.03%) and trabecular BMD (positive), trabecular separation (negative), trabecular number (positive), and trabecular number standard deviation (negative). 3D plots showed a possible interaction between 25(OH)D and mean A1C on trabecular separation (see **Supplementary Figure S1**) whereby as the level of 25(OH)D increased, the level of trabecular separation and inhomogeneity of trabecular network decreased. This decline was steeper for higher levels of mean A1C, although not statistically significant. For both trabecular number and its standard deviation, low or high mean A1C had only a small or no modifying effect on these parameters caused by low 25(OH)D. At the radius, there was a borderline significant association between 25(OH)D and trabecular separation (negative), trabecular number (positive) and trabecular inhomogeneity standard deviation (negative).

At the tibia, diabetes duration was negatively and significantly associated with trabecular area (p=0.005) and borderline significant with total area (negative). At the radius, there was a borderline significant association between diabetes duration total area (negative), trabecular area (negative) and cortical thickness (positive).

## 4 Discussion

This cross-sectional study used HR-pQCT to compare parameters of bone microarchitecture and estimated bone strength of young adults with type 1 diabetes with those of a large normative database (32). Young adults with type 1 diabetes were shown to have low trabecular and high cortical bone geometry parameters (volumetric BMD and cortical thickness). No sex specific differences in bone geometry parameters were found. Our data further show that diabetes duration and modifiable risk factors of bone health, specifically 25(OH)D, PTH, and BMI, are associated with HR-pQCT parameters.

There are limited data evaluating bone microarchitecture using HR-pQCT in patients with type 1 diabetes, especially in young adults. In this study, we show that these young adults have low trabecular thickness in the tibia and low trabecular thickness and trabecular area, and borderline low, vBMD in the radius. These trabecular bone geometry parameters are consistent with the findings of studies involving both adults (17) and children (18–20). Furthermore, high cortical bone geometry parameters (cortical vBMD and cortical thickness) were found in the radius. By contrast, other studies have shown a reduction in cortical parameters. However, direct comparison is not possible either because of differences in age (i.e., older adults or children/adolescents) or because of small control groups (17–20).

There is increasing concern that obesity may be associated with suboptimal bone strength. However, most published studies are cross-sectional with a lack of longitudinal data to support cross-sectional findings. In this study, 47% of the cohort was either overweight (n=25) or obese (n=16) based on obesity guidelines of the Canadian Medical Association. BMI was found to be positively and significantly associated with cortical area and failure load, suggesting that BMI may have a positive impact on bone health. Our study found no association between BMI and increased cortical parameters, suggesting that obesity may not contribute to increases in cortical bone. These findings support the proposal that high cortical bone may be a compensatory response to low trabecular bone.

In the healthy population, increased cortical geometry (cortical vBMD and cortical thickness) would be expected to improve bone quality. However, in type 2 diabetes, BMD that is normal to increased is associated with increased fracture risk. Several bone-derived factors may be altered by hyperglycemia as advanced glycation end products negatively impact the extracellular matrix and bone strength. This may also be relevant to type 1 diabetes, negating any positive benefits from cortical size. Increased cortical porosity would likely reduce bone quality. Elevated cortical porosity has been described in patients with type 2 diabetes possibly associated with microangiopathy and obesity (36–39). In our cohort, cortical porosity at the tibia, although not associated with BMI, was borderline high. Bone strength as measured by failure load was normal, implying a balance between the factors promoting and preventing fractures in this population.

Vitamin D deficiency and secondary hyperparathyroidism are important in increased fracture risk (6). Osteomalacia and rickets are clinical expressions of low levels of vitamin D, which increase the risk of fracture (25, 40). Our study identified a high rate of insufficient/deficient vitamin D levels using the criteria of <50/30 nmol/l. These findings are similar to previous studies from our diabetes clinic in which a higher prevalence of vitamin D deficiency/insufficiency was found compared with healthy controls (41). Lower 25(OH)D levels were found to be significantly associated at the tibia with increased trabecular separation and lower trabecular number and higher trabecular number standard deviation, a measure of inhomogeneity of trabeculae, indicating advancing trabecular disruption. These results are consistent with previous studies evaluating 25(OH)D level and HR-pQCT in non-diabetic populations. Cheung et al. (42), using HR-pQCT, found that in healthy girls, cortical area, cortical thickness, and trabecular thickness were significantly correlated with serum 25(OH)D levels. In boys, cortical area, cortical thickness, trabecular bone volume/total volume and trabecular separation were significantly correlated with serum 25(OH)D levels. On the other hand, Boyd et al. (43) showed that overall, there was no strong association between microarchitectural parameters and 25(OH)D levels in a cohort of older vitamin D sufficient people (age 55 ± 15 years). Nevertheless, trabecular BMD and trabecular thickness at the tibia were positively associated with 25(OH)D levels. Data in this study of young T1D adults show the association of lower 25(OH)D levels with trabecular measures at the tibia more than at the radius with no apparent impact on cortical bone. The significant associations found with trabecular separation, number and trabecular number standard deviation at the tibia supports the idea that low vitamin D levels may be contributing to the trabecular changes.

Despite the high rates of vitamin D insufficiency/deficiency in this study population (44%), PTH levels were mainly within the reference range. While many studies have suggested that PTH levels start to increase at 25(OH)D levels below 70 nmol/L, individual patients with vitamin D insufficiency do not always have high PTH levels (44). Consistent with PTH’s contribution to bone turnover and regulation of bone mass, studies examining high PTH levels on bone have shown that increased PTH levels impact both cortical and trabecular compartments as well as bone strength (45). In this study we found, at the radius, a significant negative association between PTH levels and cortical bone mineral density (p<0.004) and cortical area (p<0.002). The significance of these novel and intriguing findings of an association between PTH levels, within the reference range, and HR-pQCT parameters are uncertain and not documented in the literature, requiring further study. However, the advent of secondary hyperparathyroidism with its known negative impact on cortical as well as trabecular bone, could likely disrupt the current compensated state of low trabecular and high cortical bone parameters.

Both A1C and severe hypoglycemia, have been implicated in an increased rate of fractures in type 1 diabetes, in both children and adults (1–6). Eckert et al. (46) demonstrated that even moderately poor metabolic control A1C, 8.4% [8.3–8.5], to be associated with fractures in both of these patient populations. In this study, there was a only a borderline significant association between long-term A1C levels with various trabecular parameters in the tibia. A potential interaction between 25(OH)D levels and A1C, was explored using three-dimensional plots. This showed that for trabecular separation, the combination of low 25(OH)D and high A1C, resulted in higher trabecular separation, although statistical significance was not reached. Mean % A1C over the preceding 10 years was 8.18 ± 1.03, suggesting that improved metabolic control may mitigate the impact of hyperglycemia on bone.

Despite the importance of physical activity for bone health, there is limited data examining the physical activity-skeletal health relationship in T1D. There are only a small number of studies assessing exercise and HR-pQCT in healthy subjects and none addressing this question in type 1 diabetes. In a study of healthy young men, Nilsson et al. (47) found in weight bearing limbs that several trabecular parameters were associated with the degree of current mechanical loading related to type of present physical activity. This data suggest that physical activity in T1D subjects might be expected to have a positive benefit on trabecular microstructure in the tibia. A moderate increase in physical activity was found in our subjects compared with the recommendations for age, but no association between physical activity as measured by Physical Activity Characteristics Questionnaire, and trabecular measures was found. However this assessment was undertaken as an exploratory analysis. Given that the gold standard for measurement of physical activity, accelerometery which provides precise real-world data regarding intensity, duration and frequency of daily activity was not used in this study, it remains possible that exercise may mitigate some of the negative effects of diabetes on trabecular bone and further studies are required.

This study has limitations and strengths. Previous fracture history was acquired using an unvalidated questionnaire and not confirmed using X-ray reports. Sex steroid measurements were not obtained at time of study. Furthermore, both physical activity and diet were captured using a questionnaire, which although validated, did not capture the long-term impact of these potentially confounding variables. Additionally, the Z-scores were based on a reference population 18 years and older, and although our study population had a few 17 year olds, it is not expected that the reference data would be much, if at all, affected. Nonetheless, the strength of this cross-sectional study is that the cohort, which has been followed for over 10 years, is well characterized and the comparison of HR-pQCT measures with a normative data base to generate Z-scores that account for matching of age, sex, and skeletal site with a similar ethnicity distribution.

In summary, our data show early evidence of trabecular and cortical microarchitectural values indicative of impairment of bone health although failure load was normal, possibly due to compensation of the cortical density and thickness. Low levels of vitamin D were also found to be common and to be associated with trabecular parameters more in the tibia than the radius with a possible synergy with A1C. The lack of significant associations with a long-term A1C of 8.18 suggests that metabolic control at this level may mitigate the negative impact of hyperglycemia on bone. BMI appears to have a protective influence and not to explain those cortical parameters that were increased. Our findings suggest that correction of low vitamin D levels, monitoring and treatment of secondary hyperparathyroidism, and optimal metabolic control may reduce the risk of fractures in the long term as this population ages.

## Data availability statement

The raw data supporting the conclusions of this article will be made available by the authors, without undue reservation.

## Ethics statement

The studies involving human participants were reviewed and approved by The Hospital for Sick Children Research Ethics Board, REB#: 1000055749. Written informed consent to participate in this study was provided by the participants’ legal guardian/next of kin.

## Author contributions

ES contributed to the study design, literature search, data interpretation, writing the manuscript and critically reviewing the manuscript. MD contributed to data collection and writing the manuscript. RV contributed to data interpretation. AS contributed to data interpretation. YE contributed to data collection, design of tables, and reviewing/editing the manuscript. RM contributed to data analysis and interpretation. FM contributed to critically reviewing and editing the manuscript. EA contributed to the dietary data collection and data interpretation. MF contributed to data collection. SB contributed to data analysis, data interpretation and editing of the manuscript. NL-T contributed to the literature search, data interpretation, design of figure and writing of the manuscript. ES is the guarantor of this work and, as such, had full access to all the data in the study and takes responsibility for the integrity of the data and the accuracy of the data analysis. All authors contributed to the article and approved the submitted version.
