# Supplementary figures and images for: Relationship between risk factors for impaired bone health and HR-pQCT in young adults with type 1 diabetes
Source: Front Endocrinol (Lausanne). 2023 Mar 3;14:1144137. doi: 10.3389/fendo.2023.1144137 (PMC10020337; doi:10.3389/fendo.2023.1144137)

TbSp : Tibia 3D plot

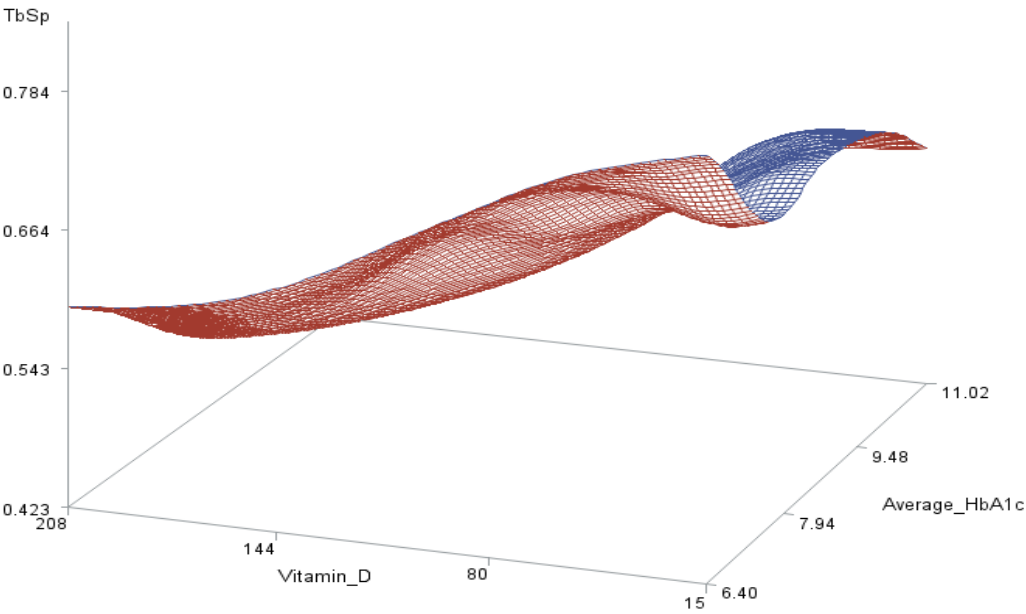

Supplement: Supplementary file 2 [file DataSheet_1.pdf]
